# Proceedings of the Canadian Thyroid Cancer Active Surveillance Study Group 2019 national investigator meeting

**DOI:** 10.1186/s40463-021-00514-0

**Published:** 2021-06-25

**Authors:** David P. Goldstein, Sangeet Ghai, Martin Corsten, Eric Bissada, Nathalie Audet, Han Zhang, Anthony Nichols, Deric Morrison, Stephanie Johnson-Obeski, Donald W. Anderson, Eitan Prisman, Nancy N. Baxter, Jennifer Jones, Amiram Gafni, Ian Witterick, Anna M. Sawka

**Affiliations:** 1 Department of Otolaryngology-Head and Neck Surgery, University Health Network, University of Toronto, Toronto General Hospital, 200 Elizabeth St., 8NU-885, Toronto, ON M5G 2C4 Canada; 2 Joint Department of Medical Imaging, University Health Network-Mt Sinai Hospital-Women’s College Hospital, University of Toronto, Toronto, Canada; 3 Division of Otolaryngology-Head and Neck Surgery, Dalhousie University, Halifax, Nova Scotia Canada; 4 Department of Otolaryngology-Head and Neck Surgery, University of Montreal, Montreal, Quebec Canada; 5 Department of Otolaryngology-Head and Neck Surgery, Laval University, Quebec City, Quebec Canada; 6 Division of Otolaryngology-Head and Neck Surgery, McMaster University, Hamilton, Ontario Canada; 7 Department of Otolaryngology-Head and Neck Surgery, Western University, London, Ontario Canada; 8 Division of Endocrinology, Western University, London, Ontario Canada; 9 Department of Otolaryngology-Head & Neck Surgery, Ottawa, Ontario Canada; 10 Department of Otolaryngology-Head & Neck Surgery, Vancouver, British Columbia Canada; 11 Melbourne School of Population and Global Health, University of Melbourne, Melbourne, Victoria Australia; 12 Department of Psychosocial Oncology, University Health Network and University of Toronto, Toronto, Canada; 13 Centre for Health Economics and Policy Analysis, Department of Health Research Methods, Evaluation and Implementation, McMaster University, Hamilton, Canada; 14 Department of Otolaryngology-Head and Neck Surgery, Sinai Health System, University of Toronto, Toronto, Canada; 15 Department of Medicine, Division of Endocrinology, University Health Network, University of Toronto, Toronto, Canada

**Keywords:** Active surveillance, Thyroid cancer, Thyroidectomy, Conference proceeding

## Abstract

**Abstract:**

Active surveillance (AS) in the management of small, low risk papillary thyroid cancer (PTC) as an alternative option to thyroidectomy, is an area of active research. A national Canadian study is proposed to evaluate the long-term outcomes of patients with small, low risk PTC who choose AS or surgery. This letter describes the proceedings of a national investigator meeting to plan the study.

**Graphical abstract:**

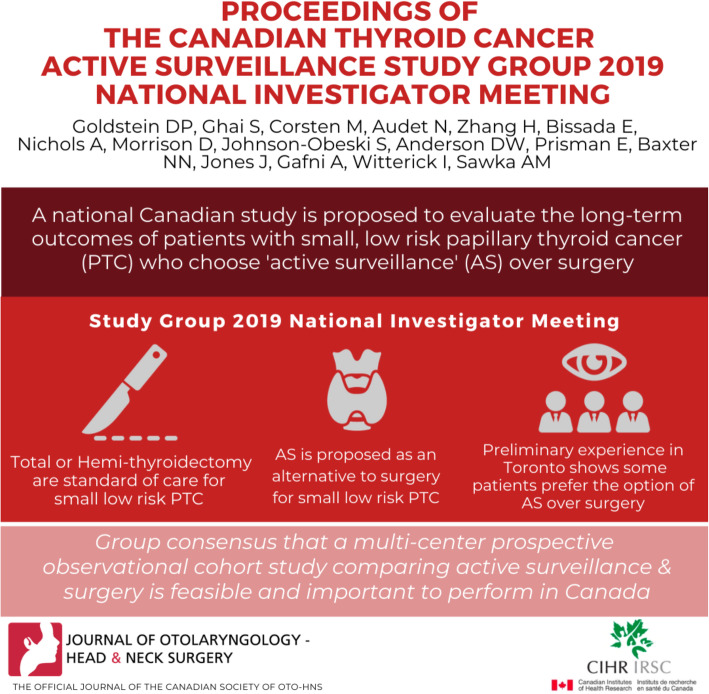

Currently about 8600 Canadians are diagnosed with thyroid cancer per year, and this number has been rising [1]. Papillary thyroid cancer, especially low risk papillary thyroid cancer (PTC), accounts for the majority of cases [2]. Although the traditional standard management of small, low risk PTC has been thyroidectomy, there is current interest in an alternative option of active surveillance (AS). AS is close clinical and diagnostic test follow-up, with the understanding that if the disease progresses, curative intent surgery would be performed. Furthermore, patients choosing to undergo AS may change their mind and have surgery at any point in follow-up, even if the disease does not progress. Cho et al. recently reported that in a recent systematic review and meta-analysis examining AS outcomes, that within a 5-year time frame, 94.7% of untreated patients did not experience significant tumor growth and 98.4% did not experience nodal metastases [3]. Furthermore, on reviewing the relevant literature and contacting experts, we found no reports of any thyroid-cancer related deaths nor distant metastases in patients with small, low risk PTC under AS [4]. Much of the existing long-term outcome literature on thyroid cancer AS describes the clinical course of patients with papillary microcarcinoma (PTC ≤1 cm) followed in Japan [5]. A prospective study offering low risk PTC patients the choice of AS or thyroid surgery (standard of care) has been conducted at University Health Network in Toronto, Canada since May of 2016, with ongoing recruitment and follow-up [6]. Eligible patients are individuals with with small (< 2 cm maximal diameter), PTCs where the disease is confined to the thyroid (i.e. no known nodal or distant metastases nor extrathyroidal extension) and the tumor is not encroaching critical structures (trachea or course of recurrent laryngeal nerve) [6].

We herein describe the proceedings of a national investigator meeting held in Toronto on November 30, 2019, to plan a pan-Canadian prospective cohort study examining outcomes of patients with small, low risk PTC who choose AS or surgery. Ethics approval was not required as this is a summary of a meeting. The pan-Canadian study would build on the findings of the original Toronto study and ultimately the results would be reported together. The 1-day meeting was sponsored by the Canadian Institutes of Health Research (Planning Grant) and the Princess Margaret Cancer Centre Endocrine Oncology Site Group. Participants included eight head and neck surgeons, two endocrinologists, one radiologist and one statistician. There were also two patient participants invited to speak (one of whom had prior thyroidectomy for PTC and another who was under AS). The format of the scientific program was that of formal lectures with subsequent discussion. The patient participants were interviewed by a meeting host in presence of the investigators and following the interviews, the investigators had the opportunity to ask the patients’ questions. The patient participants discussed their thyroid cancer care experiences and shared insights on what they felt was important for doctors/investigators to know in caring for thyroid cancer patients. The Toronto co-primary investigators (Anna Sawka and David Goldstein) hosted/organized the meeting, moderated the discussions, and took written meeting notes. The topics discussed, all relating to low risk PTC, included: a) Review of the current experience in AS of low risk PTC – worldwide and in Canada (Toronto), b) Diagnostic imaging considerations in AS, c) Presentation and feedback on a proposed national study protocol with discussion of feasibility considerations, d) Statistical design/sample size considerations, e) Patient perspectives (interview of 2 individuals), and f) Ideas for possible future ancillary studies. The key concepts distilled from the meeting by the co-primary investigators are shown in Table 1. In concluding the meeting, the group strongly agreed that a multi-center prospective observational cohort study comparing AS and surgery for small, low risk PTC is feasible to perform in Canada. Furthermore, the participants agreed to work together in conducting such a study, across multiple Canadian institutions (operating funds for the initiation of this pan-Canadian study secured from the Canadian Institutes of Health Research and the Canadian Cancer Society).

**Table 1:** Key concepts at from a Canadian national investigator meeting regarding research on AS for low risk PTC

1. Currently in Canada, patients with small, low risk PTC, are typically referred for consultation on thyroid surgery (total or hemithyroidectomy) and this is established standard of care in our country.	
2. Active surveillance (AS) of low risk papillary microcarcinoma (PTC ≤1 cm in maximal diameter) appears safe, based on the published literature (most of which is from Japan). More prospective long-term outcome research is needed regarding AS of larger low risk PTCs (i.e. primary tumors > 1 cm but < 2 cm).	
3. Preliminary experience from Toronto, suggests that some Canadians with small, low risk PTC prefer the option of AS, over thyroid surgery, if given the choice. Patient participants at the meeting strongly valued patients being fully informed about both options, and the opportunity for patient involvement in medical decision-making.	
4. Prospective AS research in low risk PTC should include patient-centered outcomes such as: disease progression requiring surgery (for patients under AS), disease persistence or recurrence requiring treatment (for the surgical arm), and validated measures of health-related quality of life and relevant related symptoms (such as anxiety).	
5. Standardization of neck ultrasound interpretation and reporting, particularly with respect to eligibility criteria and criteria for disease progression, is of critical importance in conducting AS research for PTC.	
6. A multi-center prospective observational cohort study comparing AS and surgery for small, low risk PTC is feasible to perform in Canada given the frequency of diagnosis, the availability of necessary technology and expertise, and a strong interest among clinicians to mitigating treatment-related harm in patients who may not benefit from surgery.	
7. It will be important to compare the healthcare resource implications over time, of AS compared to surgery for low risk PTC, using Canadian healthcare costs.	

## Data Availability

Not applicable.

## Abbreviations

PTC: Papillary thyroid cancer
AS: Active surveillance
